# Artifact characterization and mitigation techniques during concurrent sensing and stimulation using bidirectional deep brain stimulation platforms

**DOI:** 10.3389/fnhum.2022.1016379

**Published:** 2022-10-19

**Authors:** Michaela E. Alarie, Nicole R. Provenza, Michelle Avendano-Ortega, Sarah A. McKay, Ayan S. Waite, Raissa K. Mathura, Jeffrey A. Herron, Sameer A. Sheth, David A. Borton, Wayne K. Goodman

**Affiliations:** ^1^Brown University School of Engineering, Providence, RI, United States; ^2^Department of Neurosurgery, Baylor College of Medicine, Houston, TX, United States; ^3^Menninger Department of Psychiatry and Behavioral Sciences, Baylor College of Medicine, Houston, TX, United States; ^4^Department of Neurological Surgery, University of Washington, Seattle, WA, United States; ^5^Department of Veterans Affairs, Center for Neurorestoration and Neurotechnology, Rehabilitation R&D Service, Providence, RI, United States

**Keywords:** deep brain stimulation, implantable devices, artifact characterization, bidirectional platforms, neuromodulation

## Abstract

Bidirectional deep brain stimulation (DBS) platforms have enabled a surge in hours of recordings in naturalistic environments, allowing further insight into neurological and psychiatric disease states. However, high amplitude, high frequency stimulation generates artifacts that contaminate neural signals and hinder our ability to interpret the data. This is especially true in psychiatric disorders, for which high amplitude stimulation is commonly applied to deep brain structures where the native neural activity is miniscule in comparison. Here, we characterized artifact sources in recordings from a bidirectional DBS platform, the Medtronic Summit RC + S, with the goal of optimizing recording configurations to improve signal to noise ratio (SNR). Data were collected from three subjects in a clinical trial of DBS for obsessive-compulsive disorder. Stimulation was provided bilaterally to the ventral capsule/ventral striatum (VC/VS) using two independent implantable neurostimulators. We first manipulated DBS amplitude within safe limits (2–5.3 mA) to characterize the impact of stimulation artifacts on neural recordings. We found that high amplitude stimulation produces slew overflow, defined as exceeding the rate of change that the analog to digital converter can accurately measure. Overflow led to expanded spectral distortion of the stimulation artifact, with a six fold increase in the bandwidth of the 150.6 Hz stimulation artifact from 147–153 to 140–180 Hz. By increasing sense blank values during high amplitude stimulation, we reduced overflow by as much as 30% and improved artifact distortion, reducing the bandwidth from 140–180 Hz artifact to 147–153 Hz. We also identified artifacts that shifted in frequency through modulation of telemetry parameters. We found that telemetry ratio changes led to predictable shifts in the center-frequencies of the associated artifacts, allowing us to proactively shift the artifacts outside of our frequency range of interest. Overall, the artifact characterization methods and results described here enable increased data interpretability and unconstrained biomarker exploration using data collected from bidirectional DBS devices.

## Introduction

The recent expansion of deep brain stimulation (DBS) technologies has enabled unique opportunities to record intracranial neural activity during concurrent stimulation (Stanslaski et al., 2012; Gilron et al., 2021). One example of such an implantable neural stimulator (INS) is the Medtronic Summit RC + S (Herron et al., 2018; Stanslaski et al., 2018). The Summit RC + S has been used extensively to record neural activity in patients with neuropsychiatric disorders, enabling insights into DBS impact on symptom states (Johnson et al., 2021; Provenza et al., 2021). Not only do bidirectional systems provide opportunities for biomarker exploration in ecologically valid environments, a prerequisite for adaptive DBS, but they also allow us to better understand the underlying pathophysiology of neurological and psychiatric disorders (Gregg et al., 2021; Johnson et al., 2021; Pal Attia et al., 2021; Provenza et al., 2021). However, the artifacts introduced by high amplitude, high frequency stimulation present a challenge for analysis and interpretation of the relatively lower amplitude neural signals that we aim to measure (Zhou et al., 2018; Dastin-van Rijn et al., 2021). Therefore, it is important to identify potential artifact sources introduced by stimulation, developing techniques to mitigate these artifacts during data collection.

Previous studies have recommended optimal sense configurations for neural data collected during concurrent stimulation across several indications and stimulation targets (Ansó et al., 2022). Optimal configurations include sensing in a bipolar configuration where two contacts flank the monopolar stimulation channel, using active recharge, and blanking the amplifier during the stimulation pulse (Stanslaski et al., 2018). Additional recommendations revolve around wireless data transmission settings specific to the Summit RC + S: telemetry mode and telemetry ratio (Stanslaski et al., 2018). Telemetry mode determines the distance required between the INS and communication telemetry module to minimize data loss. Greater telemetry modes allow for increased data transmission at the expense of decreased telemetry range. Similarly, telemetry ratio values describe the proportion of uplink to downlink transmission timelines between the INS and tablet. Higher ratio values lead to slower transitions from uplink to downlink transmission, spending more time transmitting data from the INS before receiving instructions from the computer. Lower ratio values should be considered in implementation of “distributed” closed loop stimulation to decrease latency between symptom onset and stimulation changes (Herron et al., 2018).

Despite this guidance regarding the proper configuration of the Summit RC + S to collect neural data, stimulation and system-related artifacts can remain a significant problem, particularly when the SNR is very small. Furthermore, most of these artifact mitigation strategies have focused on movement disorders (e.g., Parkinson’s disease and essential tremor) applications, for which neural activity in the DBS target region is relatively large (20–100 μVrms) and DBS amplitude is relatively small (less than 2 mA) (Koeglsperger et al., 2019; Ansó et al., 2022). For example, DBS for psychiatric disorders including OCD employ high amplitude (4.5–6 mA) stimulations to the VC/VS, whereas the amplitude of target neural features are reported between 1–20 μVrms (Provenza et al., 2021; Adkinson et al., 2022; Ansó et al., 2022). The injection of high amplitude stimulation leads to a decrease in SNR, making biomarker detection more difficult (Kopell et al., 2004; Greenberg et al., 2006; Ramasubbu et al., 2018). This injection of stimulation artifact, specifically at high stimulation amplitudes, can lead to “slew overflow,” a form of signal distortion that occurs at the analog to digital converter (ADC), where the time-voltage signal changes too rapidly for the ADC to properly resolve the signal.

Here, we have characterized common artifacts that appear in response to high amplitude, high frequency stimulation for OCD. Specifically, we report data from a clinical trial for developing adaptive DBS in OCD using the Medtronic Summit RC + S (NCT04806516). First, we characterize stimulation artifact distortion due to slew overflow. Next, we identify low frequency artifacts (below the 150 Hz stimulation artifact) modulated by communication parameters. Lastly, we provide recommendations for sensing and telemetry configurations to optimize data quality, hence allowing for biomarker exploration in the entire frequency spectrum.

## Materials and methods

### Study participants and design

**FIGURE 1:**
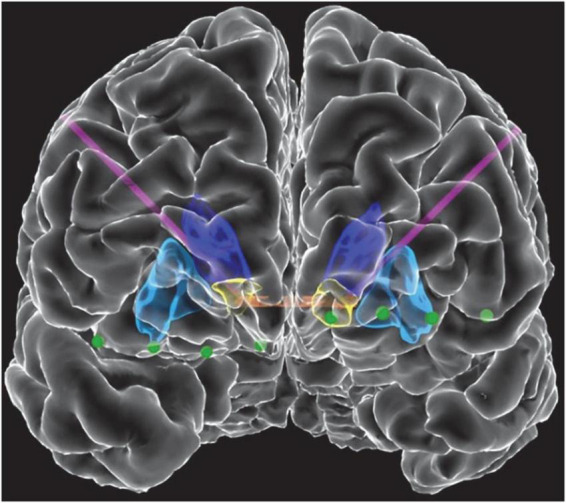
Front view of view of the reconstructed cortical surface and subcortical structures. Schematic includes DBS leads (purple) and electrocorticography contacts (green). Colored regions indicate the anterior commissure (orange), caudate (dark blue), putamen (light blue), and VS (yellow).

Three patients with medically refractory OCD were implanted with the Medtronic Summit RC + S as part of an IRB and IDE approved study. DBS leads were placed bilaterally in either ventral capsule/ventral striatum (VC/VS; Figure 1) or bed nucleus of the stria terminalis (BNST). Electrocorticography (ECoG) strips were also placed bilaterally in the orbitofrontal cortex (OFC; Figure 1), a brain region implicated in OCD symptoms of inflexibility and dysfunction of reward processing (Goodman et al., 2021). Analysis in this paper specifically highlights artifact characterization performed in P2, with a comprehensive list of artifacts and stimulation parameters for each participant listed in Table 1. Further, we include impedance measurements recorded from P2 in Table 2.

**TABLE 1:** DBS surgery targets, stimulation contact, therapeutic stimulation amplitude and telemetry settings for each participant.

Participant	P1		P2		P3	
Stimulation target	VC/VS	VC/VS	VC/VS	VC/VS	BNST	BNST
Stimulation contact	1-/C +	1-/C +	1-/C +	1-/C +	2-/C +	1-/C +
Therapeutic stimulation amplitude	5 mA	5 mA	5.3 mA	5.5 mA	4 mA	4.5 mA
Telemetry ratio	32	32	32	32	32	32
Identified stimulation artifact distortion pre-sense blank change?	Yes	Yes	Yes	Yes	N/A	N/A
Existing modulation artifacts?	Yes	Yes	No	Yes	Yes	No
Location of modulation artifacts (Hz)	27, 54, 97, 124	27, 54, 97, 124	N/A	27, 54, 97, 124	27, 124	N/A

Stimulation artifact distortion was identified at default sense blank settings. Specific locations of modulation artifacts were identified when applicable.

**TABLE 2:** Impedances recorded on left and right hemispheres from P2 within 1 month of testing.

Hemisphere	Left						Right					
Contact pair	0–1 +	0–2 +	0–3 +	1–2 +	1–3 +	2–3 +	0–1 +	0–2 +	0–3 +	1–2 +	1–3 +	2–3 +
Impedance value (Ohms)	1,628	2,330	2,560	2,008	2,280	2,913	1,968	2,838	2,878	1,983	2,123	2,765

Stimulation of the VC/VS (or BNST; P3) was performed to treat OCD symptoms, using monopolar stimulation. Per optimal configurations previously described, we used active recharge and sensed in a bipolar configuration where two contacts flanked the monopolar stimulation channel (Stanslaski et al., 2018). Neural recordings were obtained from bilateral DBS electrodes targeted to VC/VS (or BNST; P3). Recordings were bipolar, such that recording contacts in each hemisphere (contact pair 0–2) flanked the monopolar stimulation contact (contact 1). The fourth contact (contact 3) was unused. Two bipolar recording channels were also obtained from the two pairs of contacts (contact pairs 8–9 and 10–11) on both ECoG strips. The DBS electrode (Medtronic Model 3387) and 4-contact flexible ECoG paddle (Medtronic Model 5387A) in each hemisphere were connected to an implantable neural stimulator (INS), such that each DBS electrode was connected to an independent INS (de Hemptinne et al., 2021). In total, there were three bipolar recording channels per hemisphere, one sensing VC/VS activity, and two sensing OFC activity.

### Sending and transmitting neural signals

Recordings were performed at a sampling rate of 500 Hz with a high pass filter of 0.85 Hz. The low-pass filter stage 1 and 2 cutoff frequencies were both set to 100 Hz. Bidirectional communication between the INS and tablet is facilitated by the clinician telemetry module (CTM). Time-series voltage data collected onboard the device is assembled into packets and transmitted from the INS to a tablet via the Bluetooth connection established by the CTM. Similarly, stimulation parameter changes are sent from the tablet to the INS via the same CTM connection. The CTM facilitates either uplink (i.e., data sent from the INS to the tablet) or downlink transmission (i.e., data sent from the tablet to the INS), where transitioning from one direction to the other is referred to as the telemetry “ratio.” Higher ratio values indicate that more time is spent transmitting packets of data from the INS before instructions are sent from the tablet to the INS. Lower ratio values reduce the time spent transmitting packets before sending a tablet instruction, leading to a faster rate of change between data collected and instructions relayed.

Telemetry “mode” is also a configurable parameter, which is related to the range in distance allowed between the CTM and INS. A greater telemetry mode requires a shorter range in distance but enables maximum data transmission rates. For this study, telemetry mode was set to the maximum value of 4.

### Neural data analysis

Neural data analyses were performed offline, based on previous methods employed using the RC + S platform (Gilron et al., 2021; Provenza et al., 2021). LFP data were divided into 10-s segments. Any 10-s segments containing packet loss were excluded from analysis. Power spectral density estimates were calculated using pwelch in MATLAB. The mean of the entire recording was subtracted from each 10-s window to account for DC offset. A Hamming window was employed to divide each 10-s segment into 500-ms segments with 250 ms of overlap.

### Stimulation amplitude modulation testing

Amplitude testing was performed to gain insight into how high (4.5–6 mA) vs. low (less than 2 mA) amplitude stimulation impacts the quality of neural recordings. Initially, stimulation amplitudes were set to 5 and 5.3 mA for the right and left hemispheres, respectively. Amplitude changes were made in each hemisphere independently, while holding the amplitude of the opposite hemisphere constant. For example, amplitude in the left hemisphere was kept constant at 5.3 mA while amplitudes in the right hemisphere were decreased in 0.5–0.8 mA increments. Once 2 mA was reached, amplitude was then increased in 0.5–0.8 mA increments. Neural data was recorded for 1 min at each increment. In total, there were 2 min of recordings at each amplitude increment in each hemisphere. The 2–5.3 mA range was used to represent therapeutic amplitudes used in both movement disorders (∼2 mA) and psychiatric disorders (over 4.5 mA). We focus specifically on contact pair 0–2 recordings (VC/VS; P2) due to their proximity to the stimulating contact. Prior to completing testing, each hemisphere was set back to the initial therapeutic stimulation parameter settings.

### Measuring slew overflow

We quantified the percentage of neural data packets affected by slew overflow to understand the impact of high amplitude stimulation on our low amplitude recordings. Slew overflow specifically refers to when the slew rate, or the maximum rate of change over time, exceeds that measurable by the ADC. Slew overflow occurs when the stimulation amplitude is very high, such as in the case of psychiatric disorders where DBS amplitudes typically exceed 5 mA. Increasing the stimulation amplitude leads to larger rates of change in amplitude over time (larger delta). Increasing stimulation frequency also increases rate of change over time by producing more pulses per second and effectively decreasing the amount time permitted to reach the same stimulation amplitude. Therefore, surpassing the maximum delta permissible by the ADC limits its capacity to properly resolve the input signal, leading to distortion.

The Summit RC + S platform stores data in 11 JSON files, where data is transmitted as individual packets throughout a recording (Sellers et al., 2021). One of the JSON files represents the raw time domain data, where each packet contains a field called *DebugInfo*. This field indicates if slew overflow is occurring within an individual packet via a numeric value. The value refers to a binary representation (via 4 bits), indicating the sensing channel(s) for which overflow is occurring. A value of 0 indicates that there is no overflow occurring on any of the contacts for the duration of that packet whereas a value of 1 (binary representation 0001) indicates slew overflow on the first sensing channel. A value of 8 (binary representation 1000) indicates slew overflow on channel 3. Finally, when overflow is indicated on multiple channels, the *DebugInfo* field contains values above 3. For example, slew overflow on channels zero (binary representation 0001) and three (binary representation 1000) would result in binary representation 1001. This would present a numeric value “9” in the *DebugInfo* field, indicating both channel 0 and channel 3 have overflow.

### Sense blank testing

We conducted sense blank testing to observe the impact of increased sense blank time on measured slew overflow. Sense blank time represents the time sensing is suspended during the stimulation pulse, measured between when the stimulation pulse is sent and the recording resumes (Hammer et al., 2022). The minimum sense blank value is automatically set based on pulse width, and the maximum is limited to 2.5 ms to minimize loss of meaningful data. During testing, sense blank changes were made to both hemispheres simultaneously while maintaining a constant stimulation amplitude of 5 mA. Five sense blank values were tested: 0.755, 1.001, 1.5, 2, and 2.5 ms. We specifically focus on impacts of sense blank on VC/VS recordings (contact pair 0–2), recording for 1 min at each sense blank setting.

### Modulation of low frequency artifacts and impacts from ratio changes

After identifying four consistent, focal spectral peaks in the 0–125 Hz during amplitude and sense blank testing, we analyzed impacts of stimulation frequency changes on artifact location. Specifically, we aimed to understand if these artifacts were aliases of the stimulation artifact at 150.6 Hz. We tested three different stimulation frequencies, 50, 100, and 149.3 Hz, recording for 1 min at each frequency.

To further characterize these artifacts, we tested the impact of telemetry ratio on artifact frequency. Telemetry ratio values can be configured within a range of 1–32. Prior to the artifact mitigation work described here, all recordings in this study were conducted using the maximum possible ratio value (32) to maximize the amount of data sent to the tablet during open loop DBS. Telemetry parameters were modified on both INS’ simultaneously, such that data from both hemispheres was always recorded using the same ratio at any point in time. Recordings were captured for the entire 1–32 ratio range. Each recording lasted 30 s (16 min of data total).

## Results

### Increasing stimulation amplitude leads to distortion of stimulation artifact

**FIGURE 2:**
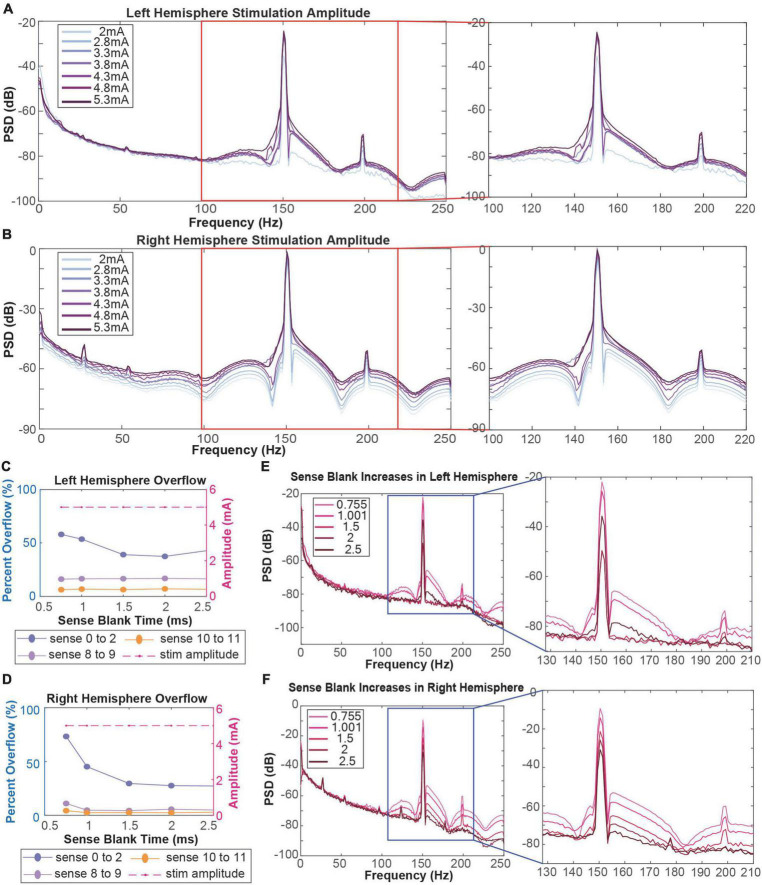
**(A,B)** Amplitude testing in the left **(A)** and right **(B)** hemispheres shows that increased amplitude leads to increased artifact distortion. Red boxes refer to stimulation artifacts and harmonics in the 130–220 Hz range. **(C,D)** Calculation of percentage of data packets with slew overflow in the left **(C)** and right **(D)** hemispheres at each sense blank value. Dotted purple lines represent data recorded in the ventral striatum. Dotted pink and orange lines represent data recorded in the orbitofrontal cortex. Dashed red line represents the stimulation amplitude at each sense blank value. **(E,F)** Sense blank impacts on left **(E)** and right **(F)** hemisphere spectral data. Blue boxes refer to stimulation artifacts and harmonics in the 130–210 Hz range.

We performed amplitude testing to understand the impact of high amplitude stimulation on the quality of neural recordings. Specifically, we conducted testing in the 2–5.3 mA range on both the left and right hemispheres, changing the amplitude in one hemisphere while keeping the amplitude in the contralateral hemisphere constant. At lower amplitudes, the left hemisphere artifact is localized to 150.6 Hz with small side lobes (Figure 2A). Further, the harmonic at ∼199 Hz is relatively small in amplitude. Increasing stimulation amplitude by ∼0.5 mA increments led to increased distortion of the stimulation artifact, producing a wider artifact bandwidth and larger side lobes. Increasing beyond 3.8 mA, the side lobes begin to subside as the artifact at 150.6 Hz becomes one large and unlocalized curve. For example, the frequency range of the localized stimulation artifact at 2 mA is ∼147–153 Hz. At the maximum amplitude of 5.3 mA, the bandwidth of the artifact itself increased by about sixfold to ∼140–180 Hz. Amplitude increases in the right hemisphere show a similar pattern where greater amplitudes lead to more stimulation artifact distortion (Figure 2B). Specifically, as amplitude is increased past ∼3.5 mA, side lobes dissipate as the stimulation artifact bandwidth increases (∼147–153 to 140–180 Hz). Contrastingly, amplitude testing in the right hemisphere showed distortion at all amplitudes, containing large side lobes around the stimulation artifact at 2 mA.

### Increasing sense blank times reduces slew overflow and artifact distortion

To mitigate slew overflow in our neural recordings, we performed sense blank testing in the 0.755–2.5 ms range on both hemispheres, simultaneously (Figures 2C,D). Initially, a sense blank value of 0.755 ms demonstrated 60 and 80% overflow in the left and right hemispheres, respectively. This percentage represents the percent of overall packets during which overflow occurred. Increasing sense blank time led to mild decreases in overflow percentage in the left hemisphere (Figure 2C), with the lowest percentage being ∼40% overflow at a sense blank time of 2 ms. The right hemisphere (Figure 2D) showed larger changes in percent overflow, decreasing to ∼30% at 2.5 ms. The dashed red line in Figures 2C,D shows that amplitude was constant throughout this testing, ensuring overflow changes were not due to amplitude changes.

Next, we assessed how sense blank changes impacted artifact distortion (Figures 2E,F). We observed that increasing sense blank time led to decreases in peak power (dB) of both stimulation artifact (150.6 Hz) and harmonic artifact (199 Hz) in the left and right hemispheres. Further, increases in sense blank time led to decreases in stimulation artifact distortion. At a sense blank of 0.755 ms, peak (dB) of side lobes were approximately −70 to −60 dB and −60 to −50 dB on the left and right hemispheres, respectively. As sense blank increased from 0.755 to 2.5 ms stimulation artifact side lobes decreased in amplitude, leaving the artifact at 150.6 Hz with a localized bandwidth of 147–153 Hz.

### Increasing amplitude increases low-frequency artifact amplitude

**FIGURE 3:**
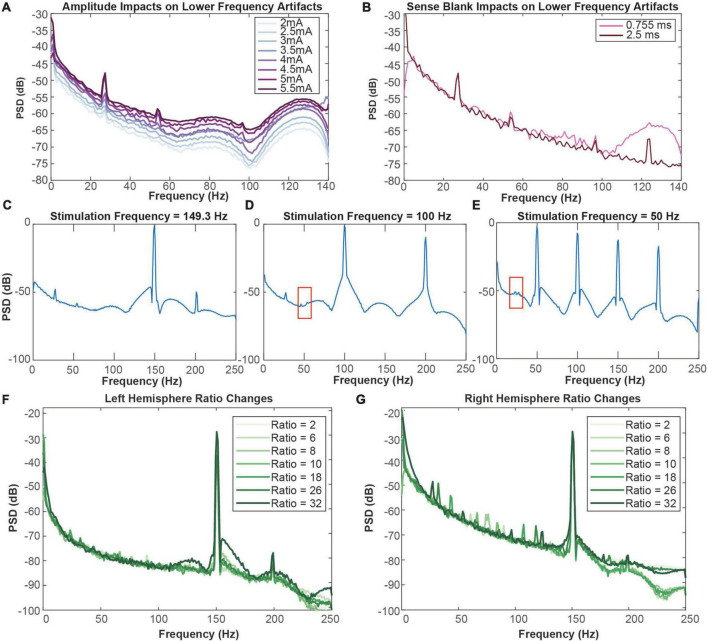
**(A)** Impact of amplitude increases on lower-frequency artifacts in the right hemisphere. The minimum amplitude tested was 2 mA (lightest) and the maximum amplitude tested was 5.5 mA (darkest). **(B)** Impact of sense blank increases on lower-frequency artifacts in the right hemisphere. The minimum sense blank tested was 0.755 ms (pink), and the maximum sense blank tested was 2.5 ms (dark pink). **(C)** 149.3 Hz, **(D)** 100 Hz, and **(E)** 50 Hz frequency testing to observe impacts on lower-frequency artifacts in the right hemisphere. Red boxes from C and E indicate areas where blockage of modulation artifacts occur due to slew overflow. **(F,G)** Representation of ratio change impacts on spectral plots (dB) across frequency (Hz). Ratio changes in the left **(F)** and right **(G)** hemispheres vary from 2 (lightest) to 32 (darkest). Blue arrows in **(G)** represent direction of artifact movement as ratio value is increased.

Throughout amplitude and sense blank testing, we observed four artifacts in the 0–125 Hz range localized at 27, 54, 97, and 124 Hz. We observed that amplitude increases led to larger artifact peaks at these frequencies (Figure 3A). From 2 to 3.5 mA, the artifacts at 54 and 97 Hz were not observed. Exceeding 3.5 mA, these two artifacts appear and increase in amplitude with each incremental ∼0.5 mA amplitude increase. Increasing sense blank time from 0.755 to 2.5 ms reduced stimulation artifact distortion, revealing a fourth artifact at 124 Hz (Figure 3B). However, the sense blank changes themselves appear to have no impact on the artifact or the frequency it resides in.

### Stimulation frequency changes demonstrate no impact on lower-frequency artifacts

To ensure the lower-frequency artifacts were not aliases of the stimulation artifact at 150.6 Hz, we altered stimulation frequency (Figures 3C–E). This testing was performed with the original sense blank value of 0.755 ms. Decreasing from 150.6 to 149.3 Hz showed no change in artifacts at 27 and 54 Hz, although 97 Hz was no longer apparent (Figure 3C). Similarly, decreasing frequency to 100 Hz still produced the artifact at 27 Hz (Figure 3D). The last frequency tested was 50 Hz, where no artifacts were distinguishable (Figure 3E). While the artifact at 54 Hz in Figure 3D and 27 Hz in Figure 3E are not as easily distinguishable due to the presence of stimulation artifact spectral lobes, some aspects of them remain (boxed in red). Therefore, it appears that stimulation artifact distortion due to slew overflow covered the 54 Hz artifact, reflecting similar mechanisms to those demonstrated with the 124 Hz artifact in Figure 3B. These do not seem to be the result of aliasing because stimulation changes result in no change to the artifact at 27 Hz. Although we tested a stimulation frequency of 100 Hz, it is clear the artifact at 54 Hz is not an alias since the expected alias would be at 50 Hz rather than 54 Hz. Overall, stimulation frequency changes have no impact on the frequency location of these lower-frequency artifacts.

### Telemetry ratio changes shift lower-frequency artifacts

We next tested impacts of telemetry ratio changes on data quality, where we specifically report analysis from ratios 2, 6, 8, 10, 18, 26, and 32 on each hemisphere. Ratio had minimal impact on left hemisphere recordings, where no lower-frequency artifacts were observed (Figure 3F). Although changing ratio occasionally demonstrated decreases in stimulation artifact peak (dB) in the left hemisphere, these changes did not seem to be related to ratio increases or decreases. Contrastingly, ratio changes in the right hemisphere appeared to shift the center frequency of the lower-frequency artifacts (Figure 3G). As the ratio increases past a ratio of 2, two artifacts appear around roughly 60 and 90 Hz. From ratio 6 to 8, the center frequencies of the artifacts seem to move toward each other and cross paths as ratio increases. Exceeding ratio values of 10, the artifacts appear to diverge. Once a ratio of 32 is reached, two additional artifacts appear. At the final ratio tested of 32 two additional artifacts appear, making four in total: 27, 54, 97, and 124 Hz.

### Amplitude, sense blank, and frequency testing shows modulation artifacts across subjects

Finally, we extensively analyzed two additional participants (P1 and P3) for both stimulation artifact distortion and lower-frequency, ratio modulated artifacts that we previously described. Results of this analysis are in Table 1, where we include information in each hemisphere on: stimulation target, stimulation contact, therapeutic amplitude of stimulation, telemetry mode and ratio, the presence of stimulation artifact distortion, pre-sense blank changes, and the appearance of lower-frequency artifacts. We define these lower-frequency artifacts as “modulation artifacts,” as their center-frequency is modulated by ratio changes. In P1, we found stimulation artifact distortion in both hemispheres resulting from slew overflow. Stimulation artifact distortion pre-sense blank increases are not present in data collected from P3, as this was a later implanted patient in which sense blank mitigation strategies had already been implemented. We also assessed the presence of modulation artifacts in P1 and P3. In P1, we identified all four artifacts in the 0–125 Hz range on both hemispheres. For P3, we identified two artifacts at 27 and 124 Hz in the left hemisphere only.

## Discussion

Evaluating and successfully mitigating all sources of artifact in neural data sensed from chronically implanted leads is a challenging task. This work builds upon our previous work on the removal of high amplitude stimulation artifacts from neural data collected onboard sensing-capable DBS devices (Dastin-van Rijn et al., 2021; Chen et al., 2022). In this study, our goal was to better understand the impact of high amplitude, high frequency stimulation on VC/VS recordings collected from bidirectional DBS platforms. The VC/VS local field potential (LFP) activity has lower peak-to-peak amplitude compared to other DBS targets used to treat movement disorders (such as STN and GPi), exacerbating already poor SNR during high amplitude stimulation. This lower peak-to-peak amplitude is common in white matter targets, which are increasingly being explored for psychiatric indications (Riva-Posse et al., 2014; Liebrand et al., 2019; Zhu et al., 2021). In this work, we characterized two previously undocumented types of artifacts in VC/VS LFP data collected in humans implanted with chronic, sensing-enabled DBS devices. We demonstrated that high amplitude stimulation leads to slew overflow and stimulation artifact distortion. Further, we discovered the presence of lower-frequency artifacts, termed “modulation artifacts,” that responded to adjusting data transmission parameters.

We first evaluated the utility of sense blanking for mitigating stimulation artifact distortion and slew overflow. Stimulation was configured for this study using active recharge, where a negative pulse is actively delivered to tissue to quickly balance the charge at the implanted electrodes. Comparatively, passive recharge relies on the post-stimulation accumulated charge at the electrode interface dissipating over time. While passive recharge was not tested in this study, it is possible that use of passive recharge would result in lower slew overflow events due to the lack of a secondary negative pulse and smaller change in charge over time as measured by the ADC. Future work should assess the potential trade-off presented by active vs. passive recharge, since the use of active recharge is promoted as a means of mitigating stimulation artifact by reducing the duration of the pulse. Overall, we found that increasing sense blank duration dramatically improved the integrity of the sensing data by reducing spectral side lobes and reducing the occurrence of logged slew overflow errors. Percentage of slew overflow and how it is represented in the neural data seemed to vary greatly, even within subject. However, it appears that certain sense blank values reflect a threshold for overflow to be represented in the neural data. Sense blank values at or exceeding 2 ms appear to minimize impacts of slew overflow on the neural data. Even though slew overflow was not observed to the same degree at these sense blank values, slew overflow was still measured at ∼40 and ∼30% for the left and right hemispheres, respectively. We recommend increasing sense blank duration to minimize stimulation artifact distortion; however, it is important to consider the tradeoff between sense blank duration and data distortion. As sense blank duration increases, the amplifier is blanked for a greater percentage of the recording. When the amplifier is blanked, the analog front end of the Summit is disconnected. Although data itself is not lost, new data points are not effectively measured during the period of sense blanking, resulting in an artifact at the frequency of stimulation. Therefore, researchers should select the minimum sense blank duration required for adequate sensing performance to minimize data distortion. The amount of distortion would be estimated by multiplying the blanking time duration by the stimulation frequency. As an example, the maximum sense blank time permissible by the Summit RC + S is 2.5 ms, which if used with 150 Hz stimulation would result in a 37.5% data distortion due to blanking time. After calculating this percentage of data loss, it was confirmed with the Medtronic engineering team as being an accurate estimate of the system behavior during blanking. It should be noted however that this data loss is not always evident in the collected data, as transient activity in analog components of the temporarily disconnected analog front end of the Summit result in time-varying samples continuing to be collected throughout the blanking period.

We also observed and documented the presence of modulation artifacts that are unrelated to stimulation artifact distortion or previously documented artifacts. However, we observed that the center-frequency of the four modulation artifacts predictably shifted when adjusting telemetry ratio. Additionally, we observed that decreases in stimulation amplitude led to decreases in the peak of each individual modulation artifact (Figure 3A). Therefore, it appears that high amplitude stimulation in low peak-to-peak neural data results in lower SNR that manifests as artifacts such as the modulation artifacts observed here. It should be noted here that DBS OFF conditions were not tested. The clinical team did not support this testing due to lack of tolerability in patients when decreasing stimulation amplitude. To mitigate modulation artifacts caused by telemetry parameters, researchers can potentially adjust the telemetry ratio and mode such that artifact peaks are outside particular bands of interest. For example, to detect biomarkers in the 0–40 Hz range, a ratio value of 10 would place modulation artifacts above 50 Hz, outside of the frequency bands of interest. However, given the observed number of peaks at frequencies which are independently variable based on selected mode and ratio, this may prove to be a challenge in some protocols. Researchers interested in analyzing broad spectral bands may find that they must adjust their classifier designs to account for the presence of these artifacts. Splitting a larger frequency band into smaller sub-bands may be problematic if using the embedded linear discriminant classifier onboard the Summit RC + S that can only use a maximum of four configured power bands. Another concern is the case of adaptive PC-in-the-loop based stimulation experiments, where the telemetry mode and ratio parameters impact the round-trip time for sensing data and commands between the PC and INS. For example, if there is an identified biomarker that changes rather rapidly in time it would be important for ratio values to be as small as possible to allow more frequent updating of stimulation parameters as symptom state evolves over time. In this context, adjusting the mode and ratio to mitigate the artifacts described in this paper may result in reduced performance of the system. Overall, these findings demonstrate the value of configurable parameters within bidirectional DBS platforms, that previously were not expected to improve the quality of neural data or closed-loop system performance.

Given the proprietary nature of Summit RC + S hardware implementation, it is unclear how these modulation artifacts emerged. It is possible that the low peak-to-peak amplitude of native neural activity in VC/VS increases the likelihood that artifacts will be introduced due to the already low SNR compared to other gray matter DBS targets. As we previously discussed, increasing stimulation amplitude worsens SNR and exacerbates the modulation artifacts. As indicated in Table 1, we observed modulation artifacts and stimulation artifact distortion to varying extents across subjects and hemispheres that cannot be explained by differences in hardware or surgical procedures. The factors driving these differences are unclear and may be due to inherent device variability during high amplitude, high frequency stimulation. In future studies, stimulation artifact distortion and modulation artifacts should be explored in a larger cohort across multiple DBS target regions, as these artifacts are patient- and target-specific. We also emphasize that future device manufacturing needs to consider how artifacts present in different ranges of brain tissue, as current knowledge is mainly geared toward gray matter targets compared to quieter, white matter regions of the brain. Another mechanism that might be attributed to the artifacts observed in this paper is the relationship between impedance changes and poor common-mode rejection. Future studies should explore common-mode rejection and how it relates data quality, with specific consideration for slew overflow and modulation artifacts. Poor common-mode rejection can occur when sensing contacts have a mismatched electrode impedance (Stanslaski et al., 2012; Tiruvadi et al., 2022). Acceptable ranges of impedance mismatch across sensing electrodes are currently ill-defined and it is unclear how minute changes in impedance impact neural data, which calls for future work analyzing the degree to which these mismatches exacerbate stimulation artifact distortion at high amplitudes. Finally, while we examined modulation artifacts and stimulation artifacts independently, we do not fully understand any potential interactions between the two, calling for future studies on these potential relationships.

Distinguishing the artifacts described in this paper from possible neural signals of interest was in many ways only possible due to the sheer configurability of the Summit system. This may be difficult in future, less configurable systems to definitively determine the nature of a potential artifact. One example is Medtronic’s Percept PC, a commercialized sensing-enabled device, where configuring sense blank values is not possible and kept at constant values. Additionally, telemetry configurations of mode and ratio are non-configurable in the Percept PC platform, resulting in potential artifacts that cannot be tracked down the same way presented here. The key takeaway is that when interpreting results from neural data, researchers should take special note of all configurable and non-configurable parameters prior to making conclusionary ties between neural signatures and behavioral outcomes, and device manufacturers should consider enabling configuration of all parameters that are known to impact neural sensing data collection.

In recent years there has been great progress in identifying and mitigating sources of artifact in neural data collected onboard sensing-capable DBS devices. When interpreting neural data or designing adaptive algorithms, it is important to better understand all potential sources of artifact that contaminate neural signals onboard bidirectional DBS platforms. Beyond the stimulation artifact distortion and modulation artifacts described in this work, it is important for researchers to also be aware of additional sources of artifact, including stimulation (Dastin-van Rijn et al., 2021; Chen et al., 2022), electrocardiogram (Neumann et al., 2021), and body movement (Thenaisie et al., 2021; van Rheede et al., 2022). While some of these artifacts may be generalizable, many may only appear in certain contexts that are device, patient, or target specific. This work represents another step in the creation of a library of expected artifacts, which we hope to continue to expand upon as more artifacts are discovered.

## Data availability statement

The raw data supporting the conclusions of this article will be made available by the authors, without undue reservation.

## Ethics statement

The studies involving human participants were reviewed and approved by the Local Institutional Review Board at Baylor College of Medicine (H-40255 and H-44941 to Baylor College of Medicine, IAA 17–27 and IAA 19–51 to Brown University, and STUDY20110082 and STUDY20110084 to University of Pittsburgh) and the US Food and Drug Administration Center for Devices and Radiological Health. The patients/participants provided their written informed consent to participate in this study.

## Author contributions

SS, DB, and WG conceived of the study. MA and NP conceptualized data analysis procedures and wrote the first draft of the manuscript. MA performed data analysis, interpreted data, and prepared figures and results with support from NP and JH. AW, MA-O, and SM performed data collection in the clinic. RM prepared the cortical reconstruction used in Figure 1. WG and SS performed clinical care aspects of the study. SS performed study surgical procedures. JH, NP, SS, DB, and WG oversaw collection of data, analysis, and manuscript completion. All authors contributed to the writing and revision of the manuscript.
